# Seroprevalence and Risk Factors Related to Bovine Brucellosis in Continental Ecuador

**DOI:** 10.3390/pathogens12091134

**Published:** 2023-09-05

**Authors:** Ana Garrido-Haro, Margoth Barrionuevo-Samaniego, Paola Moreno-Caballeros, Alexandra Burbano-Enriquez, Manuel J. Sánchez-Vázquez, Julio Pompei, Marie-France Humblet, Jorge Ron-Román, Claude Saegerman

**Affiliations:** 1 Agencia de Regulación y Control Fito y Zoosanitario—AGROCALIDAD, Quito 170184, Ecuador; ana.garrido@agrocalidad.gob.ec (A.G.-H.); margoth.barrionuevo@agrocalidad.gob.ec (M.B.-S.); paola.moreno@agrocalidad.gob.ec (P.M.-C.); alexandra.burbano@agrocalidad.gob.ec (A.B.-E.); 2 Research Unit of Epidemiology and Risk Analysis Applied to Veterinary Science (UREAR-ULg), Fundamental and Applied Research for Animals & Health (FARAH) Center, Faculty of Veterinary Medicine, University of Liège, 4000 Liege, Belgium; 3 Pan American Health Organization (PAHO), Pan American Center for Foot and Mouth Disease and Veterinary Public Health (PANAFTOSA), Rio de Janeiro 25020-000, Brazil; sanchezm@paho.org (M.J.S.-V.); juliopompei@gmail.com (J.P.); 4 Unit Biosafety, Biosecurity and Environmental Licenses, Department for Occupational Protection and Hygiene, University of Liège, 4000 Liege, Belgium; mfhumblet@uliege.be; 5 Grupo de Investigación en Sanidad Animal y Humana (GISAH), Carrera Ingeniería Agropecuaria, Departamento de Ciencias de la Vida y la Agricultura, Universidad de las Fuerzas Armadas ESPE, Sangolquí 171103, Ecuador; jwron@espe.edu.ec

**Keywords:** *Brucella* spp., cattle, Ecuador, serological survey, risk factors, competitive ELISA (c-ELISA)

## Abstract

Bovine brucellosis is a worldwide zoonotic contagious disease. According to World Animal Health Information System reports Ecuador has presented an increasing number of bovine brucellosis outbreaks in the continental territory over the past years (756 in 2018 versus 964 in 2021), generating economic losses for producers and causing a risk to public health. A cross-sectional study was conducted to investigate the seroprevalence of bovine brucellosis and associated risk or protective factors between May and June 2018. This stratified random study was implemented in 290 cattle herds located in the 23 provinces of continental Ecuador, which represents a total of 3737 cows aged 24 months or older. A competitive ELISA was used to detect *Brucella* antibodies. Simultaneously, an epidemiological survey was implemented to assess the brucellosis risk or protective factors. The apparent prevalence of bovine brucellosis at the herd level was 21.3% (95% CI: 16.8–26.6) and 6.2% (95% CI: 5.5–7) at the animal level. Univariate and multivariate logistic regression analyses were performed to determine the relationship between the potential factors associated with the presence of bovine brucellosis. The risk factors identified after multivariate analysis were a surface in ha per herd > 70 ha (OR = 2.73; 95% CI: 1.18–6.32) and the number of parturitions per animal (two or more with OR ≥ 1.8 and *p*-value ≤ 0.047). On the contrary, the protective factors were the region (farms located in the eastern region) and the absence of reported clinical signs. In addition, in herds where extensive production predominates, farmers have a low level of knowledge, and the farm biosecurity level is low. These results can guide the authorities in managing the risk factors identified, understanding the current epidemiological situation in Ecuador, improving the bovine brucellosis control program and food safety, as well as increase the one-health approach.

## 1. Introduction

Brucellosis is a zoonotic infection caused by different bacterial species of the genus *Brucella*, mainly *Brucella abortus*, *B. melitensis,* and *B. suis* [1]. It is a disease related to the evolution of agricultural society, in which animal husbandry is an integral part [2].

*Brucella abortus* is a facultative intracellular pathogen that causes persistent infection in animals; it has been isolated from several species of livestock. *Brucella abortus* is mostly associated with cattle; it is a natural or primary host, and *B. melitensis* is associated with sheep, goats, and humans [3,4]. Cattle become infected (i) after ingesting contaminated food, milk, forage, or water, (ii) through close contact with infected animals, (iii) contact with uterine secretions or aborted fetuses, (iv) by vertical, and (v) sexual transmission [3,5]. The disease causes substantial economic losses due to abortion in the last trimester of pregnancy, mastitis and reduced milk production in females, and orchitis and epididymitis in males. Infertility can occur in both males and females [6]. In humans, it is considered an occupational disease. Transmission to humans is mainly via close contact with contaminated placenta, urine, feces, blood, and aborted fetuses. Workers who handle domestic ruminants, such as veterinarians, veterinary assistants, slaughterhouse workers, butchers, as well as laboratory workers, are populations at risk [7,8].

Brucellosis has been reported in Latin America since the first decade of the 20th century and remains up to now a major zoonosis despite control campaigns. Control programs are sometimes ineffective due to the lack of sustainable funding over time [9]. The annual loss caused by bovine brucellosis was estimated at approximately $600 million in Latin America [2]. A 20–30% decrease in milk production has been estimated in brucellosis-affected herds [10,11].

The total area of Ecuador is 281,341 km^2^. It is divided into four regions, in which 24 provinces are distributed [12]. In Ecuador, the Agencia de Regulación y Control Fito y Zoosanitario (AGROCALIDAD) is the institution in charge of the national bovine brucellosis control program. That program started activities in 2008. It relies on the vaccination of females with Buck 19 and/or RB51 strains, serological diagnosis by Rose Bengal (RB), indirect and competitive ELISAs, the slaughter of positive animals, and the certification of herds as free of bovine brucellosis [13]. Certified herds are paid a bonus of USD 0.01 per liter of milk received by pasteurizers [14].

In Ecuador, the agricultural sector contributes to the gross national product by 8% [15], and 5.7 million liters of milk are produced per day at the national level, generating employment for 1,140,000 Ecuadorians [16]. According to the national cadaster of AGROCALIDAD (2020), Ecuador accounts for 4,525,183 cattle heads. Economic losses in the livestock herds of San Pedro de Suma, in the province of Manabí (Coastal area), would reach between US $1922 and 3843 per parish [17].

In Ecuador, several studies have been carried out to determine the brucellosis prevalence at the herd and animal levels, as well as to identify the risk factors associated with the disease. The first prevalence study in Ecuador was carried out in 1979, where a serological survey was conducted on 15,393 cattle heads, using the rapid plate agglutination test, within the frameworks of the National Animal Health Program (PNSA) [18]; the animal seroprevalence reached 6% (95% CI: 1.3–10) at the national level and from 1.97% to 10.62% in the Northern Highlands provinces, where entrepreneurial systems of dairy production predominate. In the coastal provinces, from 4.12% to 10.62% of animal prevalence was observed; in that area, extensive livestock production with low technological development predominates. Finally, in the Southern Highlands provinces, where most production units are small, the animal prevalence reached from 1.3% to 2.6%.

In 2014, Poulsen et al. [19] reported a 7.2% true animal prevalence (95% CI: 6.0–8.5%) in the epidemiological study conducted on *Brucella* infection in two provinces of Northern Ecuador, using the rose Bengal card antigen test (RBCT) on 2561 dairy animals. Prevalence varied by herd size and was higher in larger commercial herds.

In 2018, Carbonero et al. [20] conducted a cross-sectional study in the provinces of Azuay, Chimborazo, Cotopaxi, Manabí, Pichincha, Santo Domingo, Tungurahua, and Zamora Chinchipe; the seroprevalence at herd level was 45.1% (174/386) and 16.7% (445/2666) at the animal level. The associated risk factors were age, gender, animal health, nutritional management, type of herds, and a poor herd biosecurity level.

In 2021, Paucar et al. [21] conducted a study in small (less than 20 animals) and medium (20 to 70 animals) herds; the herd seroprevalence was 7.9% (95% CI: 6.79–9.03) and 2.2% (95% CI: 1.82–2.67) at the animal level. Their study estimated a true prevalence of 12.2% (95% CI: 7.8–17.9) at the herd level and 1.6% (95% CI: 1.0–2) at the animal level, associated with risk factors such as herd size, production types (milk, beef, or mixed), vaccination against brucellosis and presence of abortions in the herd. For the diagnosis, they used the Rose Bengal (RB) test and the sero-agglutination test (SAT)-EDTA.

For human brucellosis in the northwestern part of the country, the significant risk factors associated with seropositivity were contact with cattle, consumption of fetus and placenta (traditional Ecuadorian habit), and people with occupational cattle animal contacts. Among individuals, the overall seroprevalence was estimated at 1.88% (95% CI: 1.48–2.38), and the circulating strain was *Brucella abortus* biovar 4 [22]. Ron-Roman et al. (2012) [23] presented the first case of unilateral brucellosis–orchitis in a man from a rural community of Northern Ecuador who provided primary veterinary care in a cattle herd where he performed; the responsible pathogen was *Brucella abortus* biovar 1. According to the Ecuadorian Secretary of Public Health Surveillance (2020) [24], 45 human cases were recorded in 2019, and as of June 2020, two cases were registered; the most affected groups were people aged between 20 and 49 years old.

In Ecuador, the problem of brucellosis, as well as other animal diseases, is mainly related to the lack of microbiological and molecular identification of the causal agent, the lack of control of the antigens used for serological diagnosis, the lack of vaccine quality control, as well as the lack of a compensation system when positive animals are slaughtered [25]. An underlying problem is also the lack of support from authorities and decision-makers, which is reflected by the lack of financial resources for the national disease control program, making studies isolated, repetitive, and without high scientific value and contribution to the program.

A frequent hypothesis circulating among cattle breeders is that due to the existence of cross-reactions with other bacteria and the antibodies generated by the vaccine, many false-positive animals are slaughtered (Ron-Roman J. 2023, personal communication). The situation of bovine brucellosis in continental Ecuador is not completely updated and/or is based on indirect diagnostic tests, which does not allow the implementation of an efficient national control program based on scientific information.

The objectives of the present study are (i) the determination of bovine brucellosis prevalence in continental Ecuador and (ii) the determination of the putative risk/protective factors associated with the disease. The results of this study may be useful in developing and implementing control measures aimed at raising farmers’ awareness, making recommendations to strengthen the national bovine brucellosis control program, regulating agricultural management practices, and, ultimately, reducing the prevalence of livestock brucellosis in Ecuador.

## 2. Materials and Methods

### 2.1. Study Area

AGROCALIDAD, with the support of the Pan American Center for Foot and Mouth Disease and Veterinary Public Health of the Pan American Health Organization (PANAFTOSA/SVP-PAHO/WHO) in the framework of technical cooperation with Ecuador, conducted between May and June 2018, a serological study on bovine brucellosis in the 23 provinces of continental Ecuador. In the coastal region, samples were collected from six provinces: Esmeraldas; Manabí; Los Ríos; Guayas; El Oro; and Santa Elena. In the Highlands, samples were collected from eleven provinces: Carchi; Imbabura; Pichincha; Santo Domingo de los Tsáchilas; Cotopaxi; Tungurahua; Chimborazo; Bolivar; Cañar; Azuay; and Loja. In the east region, samples were collected from six provinces: Sucumbíos; Napo; Pastaza; Orellana; Morona Santiago; and Zamora Chinchipe.

### 2.2. Sample Size Calculation

The estimation of the sample size (herds and animals) was based on the number and distribution of cattle in 2017 (4,310,731 cattle heads older than 24 months and distributed in 277,076 herds) and the characteristics of the competitive ELISA (c-ELISA) test used (0.95 sensitivity [Se] and specificity [Sp] were considered). At the herd level, a confidence level of 0.95 was used, as well as an expected design prevalence of 0.15, with 0.05 precision. Indeed, 287 herds were sampled but rounded to 290 units. Herds were sampled based on a stratified random design so that the sample distribution followed the same structure as the animal population, according to the herd size (Table 1).

The number of animals to be sampled in each herd category was estimated considering an expected intra-herd prevalence of 10%, with a 0.05 precision and a 0.95 confidence level (Table 2).

To minimize the occurrence of false positive c-ELISA results due to the brucellosis vaccination in Ecuador, only 24-month-old female bovines were sampled (N = 3737).

### 2.3. Estimation of Herd Prevalence and Animal Prevalence

The brucellosis-apparent prevalence was calculated at herd and animal levels for each region. A herd was considered to be positive when there was at least one animal with a positive c-ELISA diagnostic test result. Prevalence was reported as the proportion of positive results (herds or animals) out of the total sample (herds or animals tested). The calculation was performed using the “epiR” and “RSurveillance” packages of the R software version 3.5.1 and 4.2.2 [26].

### 2.4. Epidemiological Survey

To determine the risk factors, the farmers filled in an epidemiological survey. This survey included 52 questions related to the herd and animal management. The survey collected information on herd identification and location, herd general data, general animal and pasture management, sanitary aspects, reproduction, pathologies, diagnostic tests for brucellosis, and samples collected (Table A1). The information collected was divided into four main categories: (i) herd identification and location (11 variables); (ii) general herd data (10 variables); (iii) general animal and pasture management (7 variables); and (iv) sanitary aspects (24 variables).

### 2.5. Sampling Method

Blood samples were collected in tubes without anticoagulant through the puncture of the coccygeal vein of each animal. The samples were transported to laboratories of the AGROCALIDAD network, maintaining the cold chain (4 to 8 °C). To extract the blood serum, samples were centrifuged for 5 min at 5000 rpm. The blood serum was stored at 4 to 8 °C until analysis at the AGROCALIDAD serology laboratories located in Tumbaco, province of Pichincha. The blood samples were collected with the prior authorization of the herd owner and did not generate any costs for them.

### 2.6. Diagnostic Tests Performed and Positivity Criteria

The c-ELISA test was the SVANOVIR^®^ *Brucella*-Ab kit for the detection of antibodies, used according to the manufacturer’s specifications. Optical density (OD) values were read at 450 nm (wavelength) on the ELISA Bio Tek micro-reader (Santa Clara, CA, USA). Positive and negative control sera from the kit were used to validate the ELISA plate result. The limits for validation criteria of the test were OD Conjugate control (Cc) 0.75–2.0, percentage of inhibition (PI) positive control 80–100, PI weak positive control 30–70, and PI negative control <30. The PI for the interpretation was considered negative if <30% and positive if ≥30%. The test Se and Sp were estimated between 0.95 and 1 [27]. PI of each sample was calculated as follows:

(1) $$PI=100-\frac{\left(OD\;samples\;or\;control\times 100\right)}{OD\;conjugate\;control}$$

### 2.7. Statistical Analysis

For the analysis of the risk factors, herds were classified according to the surface area in hectares (ha), i.e., four categories: 0–5 ha; 6–30 ha; 31–70 ha; and >70 ha, and according to the number of animals in the herd, i.e., three categories: small (1 to 20 cattle heads); medium (21 to 70 cattle heads); and large (>70 cattle heads).

The cross-sectional study allowed to point out potential risk or protective factors in the presence of positive results for bovine brucellosis by the c-ELISA test [28,29,30]. Variables considered in this study were divided into two parts, i.e., 27 variables at the herd level and 4 variables at the animal level.

First, a univariate analysis was performed, and OR with 95% confidence intervals (95% CI) was calculated for each variable analyzed (exposure factor). Then, with the variables considered as risk factors (*p*-value < 0.05), a multivariate logistic regression analysis was performed. The logistic regression model was performed in Stata SE 14.1^®^ (StataCorp LP, College Station, TX, USA). The model was progressively simplified by removing the least significant variable with a *p*-value > 0.05. The model was considered complete when it could not be further simplified without having a significant difference between the most complex and the simplest model (likelihood ratio test with a *p*-value < 0.05) [31]. The goodness of fit was assessed using the Hosmer–Lemeshow goodness-of-fit test [32]. The limit of statistical significance of the tests was defined at *p*-value ≤ 0.05 [33].

In addition, the distribution map of sampled herds with at least one seropositive animal was performed with the QGIS software, version 3.28.1 [34].

## 3. Results

### 3.1. Apparent Herd and Animal Prevalence

**Figure 1:**
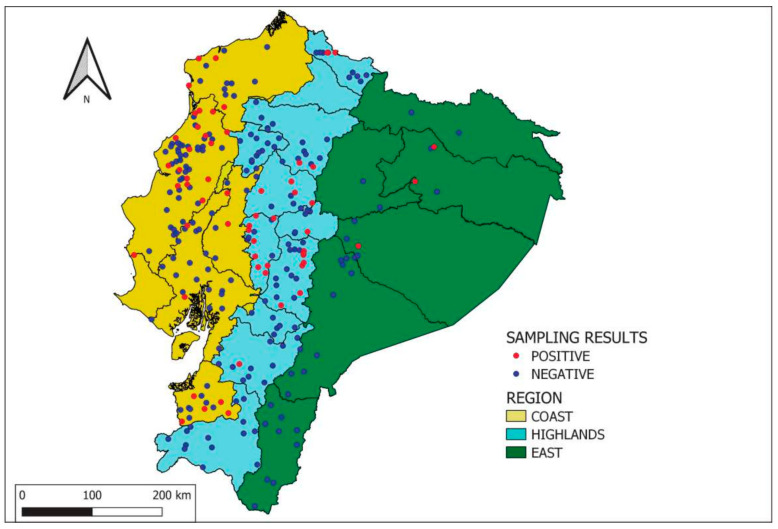
Distribution of sampled herds per region of Ecuador. Legend: positive herd if at least one seropositive animal.

The overall brucellosis-apparent prevalence at the herd level was above 20%. When considering the regions, a herd prevalence above 20% was reached in the Highlands and in the coastal region, while it was below 10% in the eastern region (Table 3 and Figure 1). The overall apparent prevalence estimated at the animal level was above 6%. The highest animal apparent prevalence, i.e., above 10%, was observed in the Highlands (Table 3).

### 3.2. Descriptive Analysis

In section I of the survey, herd identification and location, 52.4% of cattle holders indicated that they had no knowledge of animal brucellosis. In section II, general herd data, 82.4% of cattle holders implemented an extensive system, and the presence of other animals was reported in 55.5% of the herds; 19.4% of farmers reported the consumption of raw milk. In section III, general animal and pasture management, fencing was reported in 69% of the herds and containment corridors in 41.2% of the herds. Footbaths were present in 3.4% of the herds. The entry of visitors was controlled in 20.4% of herds. Paddocks were shared in 8.8% of herds. In section IV, sanitary variables, vaccination of females against brucellosis as a disease prevention measure was not performed in 89.0% of cattle herds; reproductive problems were reported in 18% of herds, the predominant signs being abortions or retained placenta.

In Ecuador, two types of vaccines are available and used for the prevention of brucellosis in cattle: the nationally-produced Buck 19 strain; and the imported RB51 biological strain. It is worth mentioning that 12.4% of cattle holders mentioned vaccination, and 5.43% of herds were vaccinated with strain 19 and 6.2% with RB51.

**Figure 2:**
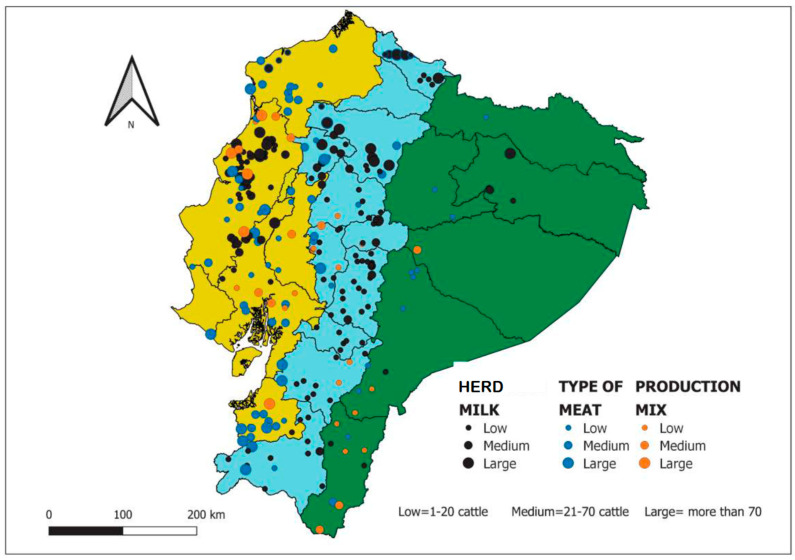
Distribution of herds according to their size and type of production.

Regarding the production characteristics, 87.9% of cattle holders reported a predominant production of meat or milk, while 12.1% of them mentioned a mixed type (meat and milk) (Figure 2). The widest reproduction mode is natural mating; it is practiced in 84.8% of herds. Extensive system is the predominant mode of farming, while intensive farming is performed in only 1.4% of herds. The animals are tied in 16.2% of herds. The presence of other animal species was mentioned for 55.5% of herds. Animal trade is practiced by 80.7% of cattle holders. However, 82.7% of them reported breeding their animals. Diagnostic tests for brucellosis are rarely performed by the farmers. Details on the variables studied by the epidemiological survey are presented in Table 4.

The coastal and Highlands regions were represented by milk cattle, with 46.27% and 83.6%, respectively. In the eastern region, 50% of herds sampled were beef holdings. More than half of herds account for 1–20 cattle heads, and most areas cover 0–5 ha.

### 3.3. Risk and Protective Variables at Herd Level

A significant correlation was found between the results of brucellosis at the herd level and for farms with a farm surface area >70 ha; therefore, the larger the herd is, the greater the risk of brucellosis, and the smaller the area, the less risk of contracting the disease. The lack of knowledge on brucellosis is a risk factor since farmers unaware of the disease are at higher risk of having brucellosis in their herd compared to those who know about brucellosis. The presence of a containment corridor in the herd appeared to be a risk factor in comparison with the herds that do not have such a facility (Table 4).

The multivariate logistic regression analysis (Table 5) confirmed the significant correlation between the results of brucellosis at the herd level and a >70 ha-farm surface area. Large herds are at higher risk of seropositivity compared to smaller ones. In addition, compared to the coastal region as a reference, the apparent prevalence was significantly lower in the eastern region.

### 3.4. Risk and Protective Variables at Animal Level

The univariate model included four variables: vaccine used; birth at the farm; clinical signs; and number of births per animal (Table 6). Registering between two and four births per animal was a risk factor for seropositivity (*p*-value < 0.05). Knowing the clinical status and the origin of the birth of animals were protective factors (*p*-value < 0.05). The multivariate logistic regression analysis at the animal level (Table 7) confirmed the significant correlation between the following factors: births at the farm; the presence of clinical signs; and the number of births per animal. It is important to mention that the animal age was not included as a variable due to the collinearity between the variables “number of births” and the “animal age”. Indeed, animals that were born on the farm or not on the farm but for which the origin was known (e.g., born on another known farm) were less likely to contract brucellosis than animals whose origin was unknown or undetermined. The absence of brucellosis clinical signs is a protective factor as well, while animals having calved at least twice were more at risk for brucellosis seropositivity.

## 4. Discussion

The agricultural sector has an important impact on Ecuador’s development, as its role is not only limited to sustaining food sovereignty but also to contributing significantly to the economy through taxes [35]. Ecuador produces 5.7 million liters of milk daily on a national scale, generating employment for 1,140,000 Ecuadorians [16].

Ecuador accounts for 4.6 million cattle heads (which represents 68% of animals in the country), distributed in three regions: 41.24% in the coastal region; 49.11% in the Highlands; and 9.65% in the eastern region. There is a total of 280,709 livestock producers nationwide [16,35]. The total income resulting from cattle farming and breeding reached USD 32,599,377 in 2019 [36].

The Ecuadorian livestock sector faces great economic losses due to different factors, among which are infectious and parasitic diseases. The economic cost of discarding each animal due to brucellosis was estimated at USD 2,217 per capita [37].

The Agencia de Regulación y Control Fito y Zoosanitario (AGROCALIDAD) has implemented a national program for the control of bovine brucellosis in the country; it is based on the following pillars: (1) vaccination of susceptible animals; (2) serological diagnosis of animals; and (3) sanitary slaughter of seropositive animals [13]. It is well known that a control program must periodically evaluate its results, with the aim to modify its actions and activities in order to achieve its objectives in an optimal term and with the least investment of resources.

One of the main factors responsible for the presence of bovine brucellosis in Ecuador is the movement of animals with unknown brucellosis status throughout the national territory or that do not comply with established sanitary requirements. Although there is currently control of animal movements within the framework of the foot-and-mouth disease eradication project [38], through the issuance and control of production and mobility certificates, to date, no operational strategy has been established for the control of brucellosis-infected animals, in violation of the article 42 of the Agricultural Health Law which focuses on “control of animal movements” [39]. In addition to the lack of control of dairy herds through the use of tests such as the ring-in-milk test (MRT) and the lack of availability of the antigen, there is no operational strategy for the direct control of infected animals in the herds [22].

In such context, this study was developed to determine the prevalence of bovine brucellosis throughout continental Ecuador (23/24 provinces), as well as to identify the possible risk factors associated with this disease. The information generated will be an input of scientific value for decision-makers and health authorities of Ecuador with respect to adjusting the national brucellosis control program.

The results found in the present study show that brucellosis is present in a great proportion of cattle herds at the country level, but especially in the coastal region and in the Highlands. In the eastern region, the prevalence appears to be lower. This updated information is in line with previous studies that highlighted a lower prevalence in the eastern region [17,18,21,25,40,41,42]. In addition, the results of herd prevalence in the Highlands and in the coastal region were not significantly different. Such observations may imply the existence of similar risk factors that favor the persistence of the disease in both regions, except climatic factors that differ in both regions, which was demonstrated in the studies of Paucar et al. (2021), Carbonero et al. (2018), and McDermott and Arimi (2002) [18,19,20,21,43].

The main difference observed with the results of Paucar et al. (2021) [21] with respect to the herd prevalence for the coastal and eastern regions could be related to the use of different diagnostic tests and possible cross-reactions with other causal agents. Indeed, such theory has already been demonstrated for Gram-negative bacteria closely related to *Brucella,* such as *Yersinia enterocolitica* O:9, *Escherichia coli* 0157:H7, *Xanthomonas maltophilia,* and *Salmonella urbana* [44]. In addition, antibodies are generated by vaccines against bovine brucellosis [45]. The Se and Sp used in the present study differ from the ones used by Paucar et al. (2021) [21]: the Rose Bengal test has 87% Se and 97.8% Sp, and the SAT test has 81.5% Se and 98.9% Sp [46]. In the present study, a c-ELISA was used, and its Sp ranges were between 99.5% and 99.6% [27]. That could minimize cross-reactions with the vaccine antibodies since it uses the M-84 monoclonal antibody specific for the polysaccharide O [47]. It is important to remember that in the present study, a herd was considered to be positive when there was at least one positive animal.

The results are also in line with reported information on the prevalence of brucellosis in neighboring countries such as Colombia, with a prevalence of 27.5% and 6.6% at the herd and animal levels, respectively [42], as well as Peru, where the prevalence results were found to be higher [1].

The univariate and multivariate analysis applied to the c-ELISA test results, in the light of the epidemiological information collected, allowed the identification of the eastern region as a herd protective factor. This observation could be explained by the climatic conditions of animal management and the apparently few movements of animals in this region [48]. Furthermore, as shown in Figure 1, the concentration of herds is lower in the eastern region, and they are mainly medium and small herds. Other studies mentioned that brucellosis prevalence was influenced by the geographic region in a country [49,50].

Another potential risk factor was a farm extending over a >70 ha-surface area. Studies by Camus (1980) and Sanogo et al. (2012) showed that the incidence of brucellosis varies proportionally with the herd size that is correlated with the farm surface area [51,52]. According to Awah-Ndukum and collaborators (2018), large herds face greater difficulties in the management of individual animals; there is often poor sanitary control generally associated with poor herd management [53]. The findings of the present investigation, as well as those presented by McDermott and Arimi (2002) [43], showed that brucellosis prevalence decreases when herd size decreases in pastoral production systems. The replacement of animals and the performance of the so-called “quarantine” are important aspects to consider in the dynamics of the disease in a herd and area; small herds generally use their own replacement animals and limit the introduction of new and potentially infected cattle [54]. Our findings are in line with that statement, as small herds have fewer seropositive animals [43,52].

At the herd level, the presence of a containment corridor was identified as a risk factor in the univariate analysis. In Ecuador, it is well known that large herds have a containment corridor, which facilitates the handling of animals, but, unfortunately, allows the contact of healthy animals with vaginal and fecal secretions of sick animals. It is important to keep in mind that brucellosis is a highly contagious disease [55], especially when considering the multiple routes and intensity of bacterial shedding. Crowded conditions during animal handling make it possible for the pathogen to spread more easily, taking into account that during an abortion, approximately 10^13^ bacteria are shed into the environment. It has been suggested that bacterial shedding at calving could infect between 60,000 and 600,000 females [56].

The univariate analysis showed a relationship between the lack of knowledge of animal brucellosis and the seroprevalence in herds. Although AGROCALIDAD implemented a national control program and brucellosis is one of the major neglected zoonoses worldwide, the Ministry of Public Health minimizes its prevalence and importance in the public health and economic sectors. Therefore, it is necessary to raise awareness of the general public and to provide training for the professionals of the livestock sector on the risks incurred by direct contact with livestock and by the consumption of fetuses and placentas (a traditional habit in Ecuador); the prevention of disease transmission is also important to avoid the spread of the infection [22]. The factors described above increase the risks for an animal to come in contact with the pathogen, especially after abortion storms, which contaminate the environment (pastures and facilities) [21,57,58]. The lack of knowledge on brucellosis has been described in several studies, so it is recommended to educate farmers urgently on the epidemiology, risk factors, and mitigation of the disease [59].

The birth of an animal on the farm itself was identified as a protective factor in the multivariate analysis, as well as the known origin of animals not born on the farm, as opposed to those whose origin is unknown. This can be explained by the fact that the animals born in the herd have an adequate epidemiological follow-up, as well as those that are formally acquired with a known origin. Additionally, this can be explained by the existence of a black market for the sale of animals that do not have the necessary authorization. The introduction of a brucellosis-infected animal in a brucellosis-free herd is a high-risk factor for the spread of the disease [60], in addition to the lack of adequate monitoring for animal movements [48].

The next variable included in the multivariate model was the number of births per animal: it was only included due to the collinearity with ages. Authors have reported the association between the cattle age and *Brucella* infection [53,61]. Age is known to be one of the factors influencing brucellosis seropositivity [62]. Indeed, the older the animal, the greater the probability of previous contact with infected animals. This is due to the lack of adequate follow-up for the elimination of positive animals in the herds [61]. Our study indicates that from the second calving onwards, there is a risk of increased exposure to *Brucella* spp., which, in some cases, may be due to a reduced immune system [63]. In Ecuador, nutritional supplementation of cattle is unusual; it is, thus, common to find cattle with advanced malnutrition; animals with poor nutritional conditions may be more susceptible to infection and a source of disease spread [20]. Other causes could be the low quality of the vaccine, a poor vaccination process, incorrect ages, wrong administration procedures, and vaccinating animals with inappropriate doses [62]. In Ecuador, two types of vaccines are available and used in cattle for the prevention of brucellosis: the nationally produced Buck 19 strain; and the imported biological RB51. Vaccinated animals had a lower risk of seropositivity than unvaccinated animals, although close to 90% of Ecuadorian farmers do not vaccinate. It is important to mention that within the framework of the national brucellosis program, vaccination is not mandatory; it is the responsibility of cattle holders to implement it [14]. That explains the poor vaccination in the herds involved in this study. Governmental agencies should take into account that incorrect vaccination and inadequate handling directly affect milk quality, as highlighted by Pacheco and collaborators (2012) [64], who determined the excretion of the B19 vaccine strain during a reproductive cycle in dairy cows [62].

One should consider that in farms where cattle holders do not perform diagnosis and elimination of brucellosis-positive animals, the risk of infection increases progressively in the herd as animals get older (permanence of animals in the herd); it was demonstrated by Ramirez et al. (2020) [42] in a study conducted in the Ecuadorian province of Manabí.

The multivariate analysis highlighted the absence of clinical signs compatible with brucellosis as a protective factor. It is important to keep in mind that, in cattle, no pathognomonic sign of the disease has been reported; the signs described vary a lot, and the disease is usually asymptomatic in young animals and non-pregnant females [1]. Given the high reproductive problem of brucellosis reported in Ecuadorian cattle [25], a study is needed to determine the causal agent of abortions because, as has been described, the high prevalence of brucellosis is related to a high incidence of abortions [50,61,65].

As for the animal management system, dairy production would favor the multiplication and spread of the bacteria within the herd [66]. In beef cattle, although animal management practices would decrease the transmission of the disease, animal holders are not very inclined to implement biosecurity measures due to the lack of perceived real benefits, which, in turn, is the case for free-ranging dairy cattle [13,67].

In the present study, regarding the farming system, herds were characterized as follows: 59.7% were dairy herds; 28.3% were meat herds; and 12.1% were a mix of both. Extensive farming was practiced by 82.4% of cattle holders. A total of 91.2% of herds shared the paddock with other animal types. It is important to point out that the predominant type of reproductive management was through natural mating, which allows the spread of brucellosis by infected males, as highlighted in previous studies [68,69].

Common other factors observed in most herds were the lack of technification, poor veterinary control, the lack of brucellosis diagnosis, and consumption of raw milk by the farmers. All these factors have also been reported in similar studies [11,21,22,25,54,57,58].

## 5. Conclusions

In Ecuador, the herd prevalence of brucellosis is high, especially in the Highlands and coastal regions (no significant difference between these two regions). That observation suggests the existence of similar risk factors, with the exception of climate, that favor the persistence of the disease. Considering the high prevalence of brucellosis in dairy herds combined with the consumption of raw milk, it is necessary to make cattle farmers, as well as the public, aware of the brucellosis transmission routes and prophylaxis measures, especially in the rural sector. Due to overcrowding and animal handling conditions, it is possible for the disease to spread more easily among animals. In Ecuador, herd vaccination coverage is low, so there is a need to raise awareness among farmers about the benefits of the proper use of vaccines in livestock, especially in high-prevalence geographic areas, to decrease disease prevalence and improve animal welfare and the quality of locally produced meat and milk. It is also recommended to carry out an adequate follow-up of animal movements, with a focus on brucellosis in accordance with the Organic Law of Animal Health. Actions to protect animal and human health should be coordinated with the Ministry of Public Health under a “One Health” strategy.

The main limitation of this study is the use of only one serological test for the diagnosis of brucellosis; applying several tests would increase the sensitivity level and reduce the proportion of false negative results [1,70,71].

## Figures and Tables

**Table 1:** Frequency distribution of herds categorized per size and distribution of cattle per herd category.

Herd Category per N Animals	N Herds per Category	N Herds Tested	Proportion of Herds (%)
≤11	180,162	60	20.7
12–24	52,848	65	22.4
25–44	25,794	58	20.0
45–98	13,893	51	17.6
>98	4379	56	19.3
Total	277,076	290	100.0

N = number.

**Table 2:** Number of females aged 24 months or older to be sampled per category.

Herd Categories per N Animals	N Cattle Heads per Herd Category	N Animals Sampled per Herd Category	Proportion of Animals per Herd Category
≤11	881,675	188	5.0
12–24	885,542	389	10.4
25–44	830,937	620	16.6
45–98	855,267	882	23.6
>98	85,731	1658	44.4
Total	3,539,152	3737	100.0

**Table 3:** Distribution of apparent prevalence per region at herd and animal levels.

Region	Herd Prevalence (95% CI)	Animal Prevalence (95% CI)
Coast Region	22.3 (15.8–30.5)	4.4 (3.6–5.5)
Highlands Region	23.7 (16.7–32.5)	10.2 (8.6–11.9)
East Region	8.8 (2.3–24.8)	1.3 (0.5–3.4)
Ecuador	21.3 (16.8–26.6)	6.2 (5.5–7)

**Table 4:** Risk and protective factors at the herd level—univariate logistic regression analysis.

Variables	Variable Type	Modalities	N Herds	N Positive Herds	N Negative Herds	Proportion of Positive Herds (%)	OR (95% CI)	*p*-Value
I. Identification and location of farm								
Region	Categorial	Coastal region	134	30	104	22.30	Ref.	-
		Eastern region	34	3	31	8.80	0.34 (0.10–1.17)	0.09
		Highlands	122	29	93	23.70	1.08 (0.60–1.93)	0.79
Cattle holder’s knowledge on bovine brucellosis	Binary	No	152	25	127	16.44	Ref.	-
		Yes	138	37	101	26.81	1.86 (1.05–3.29)	0.03
II. General data on the farm								
Surface area (ha) ^a^	Categorial	First quartile (0–5)	74	12	62	16.21	Ref.	-
		Second quartile (6–30)	77	12	65	15.58	0.95 (0.40–2.28)	0.92
		Third quartile (31–70)	67	15	52	22.30	1.49 (0.64–3.47)	0.35
		Fourth quartile (>70)	70	22	48	45.80	2.37 (1.08–5.39)	0.03
Herd size (N cattle heads)	Categorial	Low (1–20)	167	28	139	16.76	Ref.	-
		Medium (21–70)	80	22	58	25.00	0.52 (0.24–1.14)	0.10
		Large (>70)	43	12	31	27.90	0.98 (0.43–2.24)	0.96
Type of farming (production) N	Categorial	Milk	173	40	133	23.12	Ref.	-
		Meat	82	17	65	20.70	0.87 (0.46–1.65)	0.67
		Mixed	35	5	30	14.28	0.55 (0.20–1.52)	0.25
Farming system	Categorial	Extensive	239	56	183	23.43	Ref.	-
		Intensive	4	1	3	25.00	1.09 (0.11–10.68)	0.94
		Tied to the stake	47	5	42	10.60	0.39 (0.15–1.03)	0.06
Breeding system	Categorial	Artificial insemination	26	8	18	30,76	Ref.	-
		Mixed	18	6	12	33.33	1.13 (0.31–4.07)	0.86
		Natural mating	246	48	198	19.50	0.55 (0.22–1.33)	0.18
Level of technification ^b^	Categorial	High	70	17	53	24.28	Ref.	-
		Low	206	43	163	20.87	0.82 (0.43–1.56)	0.55
		Medium	12	2	10	16.60	0.62 (0.12–3.13)	0.57
Other animal species in the herd	Binary	No	129	30	99	23.25	Ref.	-
		Yes	161	32	129	19.80	0.82 (0.47–1.44)	0.49
Number of milking per day ^c^	Categorial	One fold	197	44	153	22.30	Ref.	-
		Two fold	63	14	49	22.20	0.99 (0.50–1.97)	0.99
Consumption of raw milk by the farmer ^d^	Binary	No	233	52	181	22.31	Ref.	-
		Yes	56	20	46	35.71	0.76 (0.36–1.60)	0.47
III. General management of animals and pastures								
Presence of fences in the farm	Binary	No	90	14	76	15.55	Ref.	-
		Yes	200	48	152	24.00	1.71 (0.88–3.30)	0.11
Presence of footbath	Binary	No	280	62	218	22.14	Ref.	-
		Yes	10	0	10	0.00	0.17 (0.01–2.88)	0.22
Control of visitors at the entrance	Binary	No	231	50	181	21.64	Ref.	-
		Yes	59	12	47	20.33	0.92 (0.46–1.87)	0.83
Presence of a containment corridor in the farm ^e^	Binary	No	170	28	142	16.47	Ref.	-
		Yes	119	34	85	28.57	2.03 (1.15–3.58)	0.02
Shared pasture ^f^	Binary	No	259	55	204	21.23	Ref.	-
		Yes	25	6	19	24.00	1.17 (0.45–3.07)	0.75
Mode of animal watering	Categorial	Slope	4	0	4	0.00	Ref.	-
		Rain	136	30	106	22.05	2.58 (0.13–49.21)	0.53
		River and/or ditch	99	24	75	24.24	2.92 (0.15–56.19)	0.48
		Well and/or drinking water	51	8	43	15.68	1.76 (0.09–35.78)	0.71
IV. Health aspects								
Calving place ^g^	Categorial	Corral	17	4	13	23.52	Ref.	-
		Farrowing pen	11	4	7	36.36	1.86 (0.35–9.79)	0.47
		Paddock	260	53	207	20.38	0.83 (0.26–2.66)	0.76
Disinfection of the calving place	Binary	No	254	55	199	21.65	Ref.	-
		Yes	36	7	29	19.44	0.87 (0.36–2.10)	0.76
Reproductive disorders	Binary	No	238	49	189	25.92	Ref.	-
		Yes	52	13	39	25.00	1.29 (0.64–2.59)	0.70
Number of clinical sign(s)	Categorial	No	230	49	181	21.30	Ref.	-
		One	35	7	28	20.00	0.92 (0.38–2.24)	0.86
		Two	9	3	6	33.30	1.85 (0.45–7.65)	0.40
		Three	16	3	13	18.75	0.85 (0.23–3.11)	0.81
Past brucellosis testing in the herd	Binary	No	264	55	209	20.83	Ref.	-
		Yes	26	7	19	26.92	1.40 (0.56–3.50)	0.47
Participate to the trade of animals	Binary	No	56	10	46	17.85	Ref.	-
		Yes	234	52	182	22.22	1.31 (0.62–2.78)	0.48
Origin of purchase ^h^	Categorial	Born in the herd	238	50	188	21.00	Ref.	-
		Trader	11	2	9	18.18	0.84 (0.17–3.99)	0.82
		Market or exhibition plan	24	7	17	29.16	1.55 (0.61–3.94)	0.36
		Neighbouring area	15	3	12	20.00	0.94 (0.26–3.46)	0.93
Testing at purchase ^i^	Binary	No	259	56	203	21.60	Ref.	-
		Yes	12	3	9	25.00	1.21 (0.32–4.61)	0.78
Control of the herd by a veterinarian	Binary	No	181	42	139	23.20	Ref.	-
		Yes	109	20	89	18.34	0.74 (0.41–1.35)	0.33
Use of brucellosis vaccine	Binary	No	258	56	202	21.70	Ref.	-
		Yes	32	6	26	18.75	0.83 (0.33–2.12)	0.70

Legend: OR = odds ratio; N = number; CI = confidence interval; Ref = reference. ^a^ There is no information on the surface in ha of two herds, while there is information on the number of animals. ^b^ There is no information available from the two herds on the level of technification. ^c^ There is no milking information available for 30 herds. ^d^ There is no information available from 1 herd for consumption of raw milk by the farmer. ^e^ There is no information available on a 1 herd to have a corridor in the herd. ^f^ There is no information available from the six herd shared pastures. ^g^ There is no information available from the two herds for places of calving on the herd. ^h^ There is no information on the origin of the purchase of the two herds. ^i^ There is no information on 19 herds for brucellosis testing at the purchase.

**Table 5:** Risk and protective factors associated with the seroprevalence of bovine brucellosis at herd level—multivariate logistic regression analysis.

Variable	Variable Type	Modality	OR (95% CI)	*p*-Value
Region	Categorial	Coastal region	Ref.	-
		Eastern region	0.22 (0.05–1.00)	0.05
		Highland region	1.37 (0.19–0.43)	0.33
Surface area	Categorial	First quartile (0–5 Ha)	Ref.	-
		Second quartile (6–30 Ha)	1.27 (0.51–3.17)	0.61
		Third quartile (31–70 Ha)	2.38 (0.94–6.02)	0.07
		Fourth quartile (>70 Ha)	2.73 (1.18–6.32)	0.02

Legend: OR = odds ratio; CI = confidence interval; Ref = reference.

**Table 6:** Risk and protective factors associated with bovine brucellosis seroprevalence at animal level—univariate logistic regression analysis.

Variable	Modality	N Animals	N Positive Animals	N Negative Animals	Proportion of Positive Animals (%)	OR (95% CI)	*p*-Value
Vaccine used	Both	18	0	18	0.00	Reference	-
	strain 19	390	14	376	3.58	1.43 (0.08–24.83)	0.810
	No	2900	205	2695	7.07	2.82 (0.17–46.00)	0.470
	RB51	429	16	413	3.72	1.48 (0.09–25.58)	0.790
Born on the farm	Not determined	50	8	42	16.00	Reference	-
	No	333	25	308	7.50	0.43 (0.18–1.01)	0.050
	Yes	3354	202	3152	6.02	0.34 (0.16–0.73)	0.006
Presence of clinical signs compatible with brucellosis	Not determined	73	15	58	20.54	Reference	-
	No	3638	219	3419	6.01	0.25 (0.14–0.44)	<0.001
	Yes	26	1	25	3.80	0.16 (0.019–1.24)	0.080
Number of calving per animal	0	425	16	409	3.76	Reference	-
	1	971	47	924	4.84	1.30 (0.73–2.32)	0.370
	2	818	58	760	7.09	1.95 (1.11–3.44)	0.020
	3	668	51	617	7.63	2.11 (1.19–3.76)	0.010
	4	400	31	369	8.40	2.15 (1.16–3.99)	0.020
	≥5	441	31	410	7.02	1.93 (1.04–3.59)	0.037
	Not determined	14	1	13	7.14	1.97 (0.24–15.97)	0.530

Legend: OR = odds ratio; CI = confidence interval.

**Table 7:** Risk and protective factors associated with bovine brucellosis seroprevalence at animal level—multivariate logistic regression analysis.

Variable	Modality	OR (95% CI)	*p*-Value
Vaccine used	Both	Reference	-
	Strain 19	1.45 (0.08–25.27)	0.800
	No	2.72 (0.16–45.51)	0.490
	RB51	1.30 (0.074–22.62)	0.860
Born on the farm	Not determined	Reference	-
	No	0.38 (0.16–0.91)	0.030
	Yes	0.31 (0.14–0.66)	0.002
Presence of clinical signs compatible with brucellosis	Not determined	Reference	-
	No	0.22 (0.12–0.39)	< 0.001
	Yes	0.18 (0.032–1.06)	0.058
Number of calving per animal	0	Reference	-
	1	1.19 (0.67–2.13)	0.550
	2	1.81 (1.03–3.19)	0.039
	3	2.03 (1.14–3.60)	0.016
	4	1.99 (1.07–3.69)	0.029
	≥5	1.87 (1.01–3.46)	0.047
	Not determined	2.56 (0.44–14.85)	0.300

Legend: OR = odds ratio; CI = confidence interval.

## Data Availability

The data that support the findings of this study are available from the corresponding author upon reasonable request.

## Appendix A

**Table A1:** Sampling Form and Description of Variables.

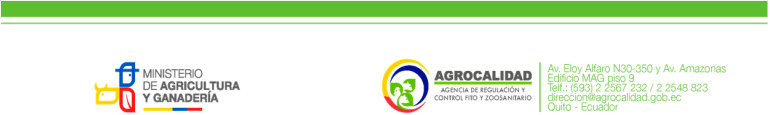

**Sampling Form to Estimate the Prevalence of Bovine Brucellosis in Continental Ecuador, 2018**								
I. Identification and Location of the Farm								
1. Date (day/month/year):				2. Name of respondent:				
3. Owner’s name:				4. Cadastral code:				
5. Province:			6. Canton:			7. Parish:		
8. Spindle or zone:		9. X:		10. Y:				
11. Do you know or have you ever heard of brucellosis:								
II. General Data on the Farm								
12. Surface area of the farm:			ha					
13. Meat cattle—number of heads					14. Dairy cattle—number of heads			
15. Type of production:				16. Number of milkings per day.				
17. Technification level:				18. Consumption of raw milk:				
19. Destination of the milk/cheese:				20. Number of cows:				
21. Presence of animals of other species on the farm:								
III. General Management of Animals and Pastures								
22. Does it have enclosures?					23. There are footbaths on the farm:			
24. Control of the entry of people:					25. Does it have a sleeve, funnel or similar other:?:			
26. Origin of the animals:					27. Do other animals occupy their paddocks?:			
28. Origin of drinking water for animals:								
IV. Health Aspects								
29. Has veterinary assistance:				30. Frequency of veterinary visits:				
31. Market your animals:				32. What category does it market:				
IV.I Vaccination								
33. Vaccination against brucellosis?				34. What vaccine applied?				
35. Who performs the vaccination?:								
IV.II Reproduction								
36. Reproduction system:					37. Do you use a specific place for births?:			
38. Do you disinfect these places?:								
IV.III Pathologies (occurring in the last year) Brucellosis								
39. Has there been reproductive problems in the last 2 years?:								
40. Symptomology presented (3 options)?:								
43. Destination of aborted material								
IV.IV Brucellosis Diagnostic Tests								
44. Admission with laboratory diagnosis:								
45. Have you carried out brucellosis tests in your herd?:								
46. Name of surveying veterinarian(s):								
V. Samples Collected								
**No.**	# Earring	AGE (Months)	Vaccine	Born on the Farm	Calving (Number)	Clinical Sign		
47								
**Location and general data:** From column A to column K, you will find the data described below: - NUMBER: default code for each sampled herd; - DATE of sampling; - RESPONDENT: name and surname of the person who was present during sampling and provided the information; - OWNER: name and surname of the farm owner; - CADASTRAL CODE: number assigned according to the cadastre during the FMD vaccination (2017 cadastre); - Province, canton, parish: spatial location of the farm, according to the geographical distribution of the country; - Herd GPS data: georeferencing of farm location on a spatial plane, including (axis, X, Y). **The data from columns:** L to BI correspond to the questions of the epidemiological survey carried out during the on-site sampling: - Do you know or have you ever heard of brucellosis: it indicates that it is a reproductive disease whose important sign to detect is abortion, so select affirmatively or negatively according to the respondent’s answer; - Area of the farm: the area corresponding to the premises and pastures, in hectares, determined in the survey, must be specified; - Meat-reared animals: indicates the number of animals that are farmed for fattening; - Dairy animals: the number of animals that are farmed for milk production (it does not matter if the animal, after its productive life, is for meat consumption destined for the slaughterhouse); - Meat and milk percentage: based on the parameters of animals intended for meat and milk, this parameter is considered; - Production: select the option according to the following conditions: ∘ Extensive: grazing is the only type of feeding; ∘ Intensive: balanced feed of vegetable origin and pastures in stables are the main types of feeding in the herd; ∘ Hogueo: cattle tied to graze in a specific place, preferably under a tree; - Type of production: refers to the type of farming: ∘ Meat: animals are farmed for fattening and selling; ∘ Milk: animals farmed for the production of milk and by-products; ∘ Mixed: animals farmed for both meat and milk production; **Number of milkings per day:** enter the number of milkings per cow and per day (maximum ); **Technification level:** select the option according to the following conditions, depending on the level of technification: - Low: manual milking; - Medium: it is carried out in hygienic conditions from the udder to a cooling tank; - High: use of milking machines; ∘ Consumes raw milk: it must be selected if the milk is consumed raw by farmer and relatives, with nopasteurization; ∘ Destination of the milk/cheese: select the option according to the following conditions, depending on the final destination of milk and by-products collected on the herd: ▪ Collection centres: they gather the milk production or by-products of small holdings so that they can compete in quantity and quality in the markets of large urban centres; ▪ The milk industry: the milk is sold to industries for further processing in the manufacture of dairy by-products; ▪ Merchant: after milking, the milk is sold to a person outside the herd who collects from various herds for final sale; **The number of cows:** indicates the number of cows (females older than 24 months) in the herd; **Presence of other animal species on the farm:** select affirmatively or negatively the existence of other animal species present on the farm; **Has fences:** select affirmatively or negatively the existence of fences; **There are footbaths on the farm:** select the affirmative or negative the existence of footbaths at the entrance to premises; **Control of the visitors:** select affirmatively or negatively the existence of a register forvisitors; **It has a sleeve, funnel or similar other:** select affirmatively or negatively the existence of places for cattle containment; **Origin of animals:** select the option according to the following conditions, depending on the origin of animals in the herd: - Farm itself: animals are born, grown, and bred in the farm itself; - Neighbour: animals were purchased from a nearby farm; - Commercial fair: animals were purchased at a commercial fair; - Exhibition fair: animals were purchased at an exhibition fair; - Merchant: animals were purchased from a cattle trader; **Other animals graze on their pastures:** select affirmatively or negatively if other livestock animals graze on its pastures (paddock rental modality); **Origin of the drinking water for animals:** select the option according to the following conditions, depending on the source of the drinking water, such as: - River and/or ditch: natural current of water that flows permanently and ends up in another, in a lake or in the sea, ditch or small channel that carries water, especially for irrigation; - Well and/or drinking water: deep holes made in the ground, especially to draw water from underground springs, water for human and animal consumption, that can be consumed without restriction for drinking or preparing food; - Rain: rainfalls; - Runoff: slope of a mountain or elevation of the land on any of its sides, it is a decline or place where the water runs. This is usually a sloping topographical surface, lying between high points (such as ridges, peaks, or ridges) and low points; **Has veterinary assistance:** select affirmatively or negatively, if a veterinary practitioner visits the farm; **Veterinary visit frequency**: select the option according to the following conditions, depending on the frequency of the veterinary practitioner’s visit: - Weekly: the veterinary practitioner visits the farm every week; - Fortnightly: the veterinary practitioner visits the farm every 15 days; - Monthly: the veterinary practitioner visits the farm every month; **Sales of animal:** select affirmatively or negatively, if the cattle holder sells them to external parties; **What animal category does she/he sell:** select the option according to the following conditions, depending on the age category to which the most animals sold fall into: - Calves: calves, females, aged 1 day to 6 months; - Heifers: calve, females, aged 6 months to 18 months or until it gives birth to calf; - Cows: females that have already calved, generally older than 24 months; - Males: age category that includes calves, bulls, and bullocks; **Vaccination against brucellosis:** select affirmatively or negatively, depending on whether the cattle holder vaccinates against bovine brucellosis; **Which vaccine applied**: select the option, depending on the vaccine applied to the animals: - Strain 19: smooth, gram-negative strain that has its entire lipopolysaccharide, including the “O” chain, responsible for inducing antibodies that react with antigens for diagnosis; - RB51: elaborated from a rough strain of *Brucella abortus*. It lacks the “O” chain of lipopolysaccharide; - Do not know: when the owner knows that the brucellosis vaccination was performed but does not know the strain applied; - Both: when the owner applied both strains at different times; **Who performs the vaccination**: Select the option, depending on the person who administered the brucellosis-vaccine by injection: - Cattle holder/administrator: the owner or the person in charge of the herd administers the vaccine; - Public MV: the vaccine is administered by a state veterinarian; - Private MV: the vaccine is administered by a private veterinary practitioner; **Breeding system used**: select the option according to the following conditions, depending on the breeding system in force in the herd: - Natural mating: consists of keeping the bull loose in the paddock, permanently with all the herd animals, so that it can interact freely with cows; - Insemination: assisted reproduction technique; - Mixed: when, according to the physical, economic, and environmental conditions, it is decided which technique (natural or artificial) will be applied according to the season; - Embryo transfer: artificial breeding method that consists in collecting an embryo from a cow uterus, i.e., the donor, to introduce it into another female uterus, i.e., the recipient. The embryo will continue growing and developing until delivery; **Use a specific place for calving**: select the option according to the following conditions, depending on the place where females calve: - Pasture: place aimed for farming and cattle grazing, wide limit; - Corral: generally uncovered enclosure, next to the rural houses, which are used to keep domestic livestock, within a designated area; - Farrowing pens: traditional construction dedicated to calving, generally the last trimester of pregnancy; **Disinfect these places**: select affirmatively or negatively, if the owner of cows close to parturition disinfects the specific places for parturitions; **There have been reproductive problems in the last 2 years**: select affirmatively, negatively or if you do not know (“do not know”) if there have been reproductive problems in the herd in the last 24 months; **Symptomatology presented (3 options):** select 3 relevant options presented: - Abortion: involuntary termination of pregnancy before the embryo or offspring is able to survive outside the womb; - Placental retention occurs after childbirth since the organ has not been expelled with the offspring; - Weak calf: clinically describes the calf that is born normally but is weak and slow to sit up and suckle; Affected animals progressively deteriorate and generally do not survive beyond a week of life; - Sterility: quality attributable to those biological organisms that cannot reproduce, either due to the malfunction of their sexual organs or because their gamete is defective; - Postpartum metritis: inflammation of the uterus usually due to a microbial infection that occurs during the 21 days (usually 10) after delivery. It is almost always seen after abnormal delivery or retained placenta; - Joint swelling: accumulation of fluid in the soft tissues surrounding the joint, due to its inflammation; - Epididymitis: inflammation of the epididymis, usually accompanied by redness and swelling of the scrotum; - Orchitis: inflammation of one or both testicles, often caused by a microbial infection, one of the causes of acute scrotum and azoospermia; - Anestrus: the period after calving during which cows show no behavioural signs of estrus, a state of sexual inactivity in females; **Destination of aborted material**: Select three relevant options presented: - Burial: the aborted material is buried; - Leave in place: if the aborted material is left on the site of abortion; - Food for other species: if the aborted material is fed to carnivorous species; - Garbage: if the aborted material is disposed of in garbage cans or left on vacant land to decay; **Entry with laboratory diagnosis**: select affirmatively or negatively, depending on whether animals newly-introduced in the herd are tested for brucellosis; **Have brucellosis tests been carried out in your herd**: select affirmatively or negatively if diagnostic tests for brucellosis have been carried out; **Name of the interviewer veterinary**: name and surname of the veterinarian or the person who conducted the survey; From column AZ to column BI is compiled the information on samples collected, with their respective results, according to the following parameters: - Earring: earring number of the sampled cow; - Age (months): age of the sampled cow in months; - Vaccine: type of vaccine administered to the cow; - Born on the farm: indicate affirmatively or negatively, if the sampled cow was born on the farm; - Calvings: indicate the number of calvings of the sampled cow; - Symptoms: Indicate affirmatively or negatively, if the cow has had symptoms of brucellosis in the last 24 months; **Report**: corresponds to the code of the AGROCALIDAD laboratory report from which the corresponding result was obtained; **Result**: The collected samples were analyzed using the characteristics of the laboratory test to be used for the analysis of samples (SVANOVIR^®^ *Brucella*-Ab C-ELISA), which has a 99.5%-sensitivity and a 99.6%-specificity; those with a PI ≥ 30% will be considered as positive; **PI**: percentage of inhibition obtained from the reading of optical densities in an ELISA reader, for the interpretation of the results; **Observations**: corresponds to criteria issued from the results.								
